# Clinicopathological Features, Treatment Outcome, and the Cryptococcal Antigen Latex Agglutination System Titer in Feline Cryptococcosis Treated with Amphotericin B and Fluconazole

**DOI:** 10.3390/vetsci12121211

**Published:** 2025-12-18

**Authors:** Suprawee Treekhunrungruang, Chompoonek Yurayart, Chaiyakorn Thitiyanaporn, Tassanee Jaroensong

**Affiliations:** 1 Kasetsart University Veterinary Teaching Hospital, Faculty of Veterinary Medicine, Kasetsart University, Bangkok 10900, Thailand; suprawee.tr@ku.th; 2 Department of Microbiology and Immunology, Faculty of Veterinary Medicine, Kasetsart University, Bangkok 10900, Thailand; fvetcny@ku.ac.th; 3 Department of Companion Animal Clinical Sciences, Faculty of Veterinary Medicine, Kasetsart University, Bangkok 10900, Thailand; chaiyakorn.t@ku.th

**Keywords:** amphotericin B, antigen titer, CALAS, feline cryptococcosis, fluconazole

## Abstract

Feline cryptococcosis is a common systemic mycosis that typically begins in the nasal cavity and can extend to the eyes, skin, and CNS. Objective monitoring during therapy remains limited. This study aimed to test whether serial serum cryptococcal antigen titers (CALAS) and a simple six-domain clinical score can track response, and to describe clinicopathologic trends during amphotericin B and fluconazole treatment. We retrospectively reviewed cases from 2014 to 2023 and analyzed a single-center cohort of 35 cats. Management used amphotericin B plus fluconazole for ~3–4 months, then fluconazole maintenance. Monthly assessments included CALAS (log_2_), the clinical score (0–18), and routine hematology/biochemistry; mixed-effects models tested change over time. CALAS fell (mean log_2_ 12.00 to 6.79), total leukocytes decreased (16.85 × 10^9^/L to 10.90 × 10^9^/L) with a similar neutrophil decline, alanine aminotransferase remained stable, and blood urea nitrogen/creatinine increased in some cats. The clinical score improved from 3.83 to 0.68. Seven cats developed azotemia during amphotericin B; the drug was stopped, renal values normalized in three, and four remained azotemic at the last follow-up. These findings support the use of CALAS and a simple score as practical monitoring tools and underscore the need for routine renal surveillance.

## Simple Summary

Feline cryptococcosis is a deep fungal infectious disease that often begins in the nasal cavity and can spread to other organs. Thirty-five cats with naturally occurring cryptococcosis were evaluated for 12 months after parenteral subcutaneous or intravenous amphotericin B combined with oral fluconazole for 3 months, followed by fluconazole alone as maintenance. At each visit, we measured a serum antigen marker (CALAS), checked routine blood tests, and used a simple six-domain clinical score. Over time, the antigen titers fell, white blood cell and neutrophil counts decreased, and the clinical score improved, indicating better overall health. Liver enzymes stayed stable, but BUN and creatinine rose in some cats. Seven cats developed azotemia during amphotericin B treatment; the drug was stopped, and BUN and creatinine returned to normal in three cats, but four cats had persistent changes. These findings support serial antigen testing and an easy clinical score to track response, and they highlight the need for regular kidney monitoring during therapy.

## 1. Introduction

Cryptococcosis is a systemic fungal disease caused mainly by *Cryptococcus neoformans* and *Cryptococcus gattii*. It affects both humans and animals. Cats are the most commonly diagnosed species [1,2,3,4]. The infection begins when environmental fungal spores are inhaled, colonize the respiratory tract, and then spread to other organs, including the central nervous system (CNS), ocular, and cutaneous tissues [5]. Ecological studies show that *C. neoformans* is strongly associated with pigeon droppings and soil enriched with bird feces, while *C. gattii* is more often linked with eucalyptus and other trees [6,7,8]. These fungi are distributed worldwide, but there are geographical differences. *C. gattii* is more frequent in tropical and subtropical regions, while *C. neoformans* has a broader distribution [9,10,11].

In humans, cryptococcosis is a significant opportunistic infection, especially in patients with advanced HIV/AIDS. Cryptococcal meningitis is a leading cause of death in these populations, particularly in Africa [12,13]. Cryptococcosis is also increasingly reported in immunocompetent individuals, with *C. gattii* infections being associated with severe CNS disease and high mortality [14,15,16,17]. In veterinary medicine, cryptococcosis is most commonly recognized in cats, though dogs may also be affected. Cats usually present with chronic nasal disease, nasal swelling, mucopurulent or bloody discharge, and sometimes skin, ocular, or neurological signs [7,18,19,20]. Retrospective studies and case series from Australia, California, and Asia have shown that the nasal form is most common; however, disseminated and CNS forms are also observed [7,9]. Dogs appear to develop more disseminated infections than cats [21].

Diagnosis can be made by cytology, histopathology, fungal culture, antigen detection, and molecular assays. The cryptococcal antigen latex agglutination system (CALAS) and the lateral flow assay (LFA) are highly sensitive tests widely used in both human and veterinary medicine [22,23]. Sequential monitoring of antigen titers is instrumental in cats for evaluating treatment response and detecting relapse [5,9,18,24]. Treatment requires long-term antifungal therapy. Amphotericin B, combined with flucytosine, is recommended for humans with cryptococcal meningitis, whereas fluconazole and itraconazole are commonly used in veterinary cases [25,26]. Despite treatment, prognosis is guarded when the CNS is involved, both in humans and cats [27,28,29,30,31].

Feline cryptococcosis is a globally recognized fungal disease, yet published data from Asian countries remain scarce, particularly regarding long-term treatment response and clinicopathological trends. In Thailand, retrospective studies addressing this issue are lacking, and no prior effort has comprehensively tracked changes in clinical status alongside antigen titers and bloodwork over an extended treatment course. Therefore, this study was conducted to help fill this knowledge gap. This retrospective study had two prespecified objectives. First, to describe the demographic, diagnostic, and clinical characteristics of all cats with cryptococcosis seen at the Kasetsart University Veterinary Teaching Hospital. Second, to evaluate longitudinal treatment response in a strictly defined subset with serial follow-up to 12 months (*n* = 35) using serum cryptococcal antigen titers by CALAS (log_2_), a six-domain clinical severity score (0–18), and routine clinicopathology, thereby providing a more complete picture of the clinical and laboratory progression in affected cats.

## 2. Materials and Methods

### 2.1. Study Design

The data for this study were obtained from the electronic medical records system of the Kasetsart University Veterinary Teaching Hospital. Data was collected from 1 January 2014 to 31 December 2023. The study protocol has been reviewed and approved by the Institutional Animal Care and Use Committee of Kasetsart University (IACUC; approval no. ACKU68-VET-013).

Cats were included in this study if they were diagnosed with cryptococcosis. Eligible cases were confirmed by cytology, CALAS, LFA, fungal culture, or histopathology. For the subgroup analysis of sequential treatment response, cats were required to have at least three post-treatment CALAS titer measurements and to have complete clinical and laboratory follow-up data across multiple time points within the 12-month study period.

Cases were excluded if the cat had previously received treatment at another veterinary facility or if follow-up data were incomplete due to missing CALAS titers or laboratory results. In addition, cats were excluded from statistical analysis if they had missing values at more than three of the six time points (months 0, 1, 3, 6, 9, and 12) or if concurrent serious systemic diseases significantly confounded their hematologic or biochemical profiles. These criteria ensured consistency and reliability in longitudinal statistical evaluations.

### 2.2. Specimen Sampling

Samples for the diagnosis of cryptococcosis were collected as part of the routine clinical work-up at the Kasetsart University Veterinary Teaching Hospital. All procedures were performed by or under the supervision of licensed veterinarians. The type and site of sample collection were determined based on the cat’s clinical signs, imaging findings, and suspected organ involvement.

For serologic diagnosis, cryptococcal antigen detection was performed using either CALAS (CALAS; IMMY, Norman, OK, USA) or LFA (CrAg^®^ LFA; IMMY, Norman, OK, USA), depending on test availability at the time of case presentation. Blood samples were obtained primarily from the jugular vein, as this site provides an adequate volume and facilitates repeated sampling in cats undergoing serial monitoring. When jugular venipuncture was not feasible because of patient temperament, body condition, or local disease, the medial saphenous vein was used as an alternative.

For fungal culture, samples were collected from clinically affected tissues or fluids. In cats with nasal or upper respiratory involvement, swabs were taken from nasal discharge, nasal cavities, or ulcerated nasal planum after gentle cleaning of the surrounding area. In selected cases, endoscopic sampling of nasal or pharyngeal tissues was also performed to obtain deeper or less accessible lesions. For cutaneous or subcutaneous lesions, exudate or tissue fragments were collected using sterile swabs or curettes. In cases with mass lesions (e.g., nasal, facial, or lymph node enlargement), tissue samples were obtained by incisional biopsy or excisional biopsy when clinically indicated. In cats with suspected neurological cryptococcosis, cerebrospinal fluid (CSF) was collected and submitted for fungal culture in addition to cytologic examination. All samples were transferred into appropriate transport media or sterile containers and submitted to the laboratory as soon as possible to optimize fungal recovery.

Cytologic evaluation was used as a rapid, minimally invasive diagnostic tool. Impression smears were prepared from ulcerated or surgically exposed lesions by gently pressing clean glass slides onto the lesion surface. Nasal discharge was collected using sterile cotton-tipped swabs and rolled onto slides to obtain representative material. Fine-needle aspiration (FNA) was performed on palpable masses, enlarged lymph nodes, or subcutaneous nodules using standard techniques. When deeper sinonasal structures required evaluation, endoscopically guided sampling of the nasal or pharyngeal cavity was performed to obtain cytologic material from lesions not accessible by routine swabbing. Smears were air-dried, stained with routine cytologic stains available in the hospital laboratory, and examined for the presence of encapsulated yeasts consistent with *Cryptococcus* spp., as well as associated inflammatory responses. For cats with suspected CNS involvement, CSF was collected, and cytospin preparations were made to increase the possibility of detecting low numbers of organisms.

For histology, tissue samples were obtained from masses, granulomatous lesions, or other abnormal tissues identified on physical examination, imaging, or endoscopy. Biopsy procedures included incisional or excisional biopsies, selected based on lesion size, location, and the patient’s overall condition. Tissue specimens were fixed in 10% neutral buffered formalin and submitted to a diagnostic pathology laboratory. Routine hematoxylin and eosin staining was performed.

### 2.3. Collected Data

The collected data included the cat’s age at diagnosis, sex, breed, clinical signs, FeLV/FIV infection status, CALAS titers, antifungal treatment, and blood test results. Blood parameters consisted of hematocrit (HCT), hemoglobin concentration (HGB), red blood cell count (RBC), mean corpuscular volume (MCV), mean corpuscular hemoglobin (MCH), mean corpuscular hemoglobin concentration (MCHC), white blood cell count (WBC), neutrophils, lymphocytes, neutrophil–lymphocyte ratio, monocytes, eosinophils, basophils, and platelet count (PLT), determined using a Sysmex XN-1000™ Hematology Analyzer (Sysmex, Mundelein, IL, USA). Serum biochemistry included blood urea nitrogen (BUN), creatinine, and alanine aminotransferase (ALT), which were measured using an IL Lab 650 chemistry system (Diamond Diagnostics, Holliston, MA, USA). Descriptive statistics were used to summarize all variables.

The Feline leukemia virus (FeLV) and Feline immunodeficiency virus (FIV) infection status of the studied cats was described using descriptive statistics to provide an overview of the health condition of the sample population, including the number and percentage of cats infected with FeLV, FIV, both, or neither. The diagnostic tests used included a point-of-care test kit for FeLV/FIV (a rapid immunomigration [RIM]-based method for detecting FeLV antigen [p27] and FIV antibodies, WITNESS^®^; Zoetis, Parsippany, NJ, USA), an enzyme-linked immunosorbent assay (ELISA) for detecting FIV antibodies and FeLV antigen (p27) (SNAP^®^ Feline Triple Test; IDEXX Labs, Westbrook, ME, USA), and polymerase chain reaction (PCR) for detecting FeLV provirus.

### 2.4. Composite Clinical Scoring System for Feline Cryptococcosis

A composite clinical score for feline cryptococcosis was assigned to each cat at every evaluation. The score comprised six organ-based domains: nasal/upper respiratory, CNS/neurologic, ocular, cutaneous/subcutaneous, systemic/constitutional, and lymph node involvement. Each domain was graded from 0 to 3, with 0 indicating no clinical signs attributable to cryptococcosis and 3 indicating severe, life-threatening involvement requiring intensive treatment or hospitalization. The domain scores were summed to obtain a total score ranging from 0 to 18. Clinical scores were recorded at baseline (month 0) and at follow-up visits approximately 1, 3, 6, 9, and 12 months after initiation of antifungal therapy. The specific grading criteria for each domain are summarized in Table 1.

### 2.5. Statistical Analysis

Sequential CALAS titers (log_2_-transformed) and the blood parameters were analyzed across six time points: month 0 (diagnosis), and months 1, 3, 6, 9, and 12 following treatment initiation. Before analysis, titer values were log_2_-transformed to stabilize variance and normalize data distribution.

To evaluate changes over time, a Linear Mixed Model (LMM) was used, treating month as a fixed effect and each case (patient ID) as a random effect to account for repeated measures within subjects. Tukey’s Honest Significant Difference (HSD) test was used for post hoc pairwise comparisons. All statistical analyses were conducted using IBM SPSS Statistics version 27.0, and a significance threshold was set at *p* < 0.05.

## 3. Results

**Figure 1:**
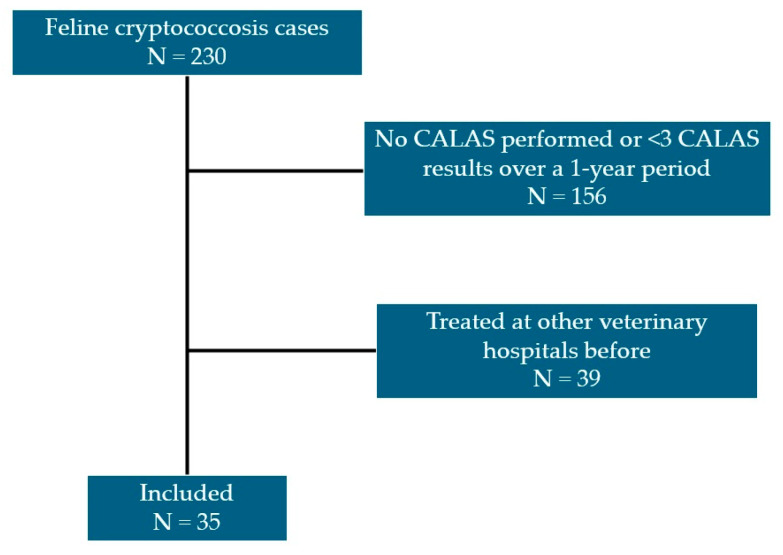
Case-selection flow diagram for cats with cryptococcosis identified from hospital medical records (2014–2023). Of 230 cats, 156 were excluded for no CALAS testing or <3 CALAS results within 12 months, and 39 for prior treatment at other veterinary hospitals, leaving 35 cats included.

This study included 230 cats diagnosed with cryptococcosis. Thirty-five cats remained eligible after exclusion (Figure 1). In 230 cats, one hundred twenty-four cats (53.9%) were female, and 106 cats (46.1%) were male. The mean age at diagnosis was 3 years and 10 months, with a range from 6 months to 16 years. Most cats were Domestic Shorthairs (218 cats, 94.8%). Other breeds included Persian (7 cats, 3.0%), Scottish Fold (3 cats, 1.3%), Abyssinian (1 cat, 0.4%), and American Wirehair (1 cat, 0.4%).

All cats were managed with a combination induction therapy consisting of amphotericin B deoxycholate (Amphotericin B; Biolab^®^ for injection 50 mg/vial; Biolab Co., Ltd., Samutprakarn, Thailand) plus fluconazole (Flucozole^®^ 50 mg capsules; Siam Pharmaceutical Co., Ltd., Bangkok, Thailand). The total cumulative amphotericin B dose per cat was recorded as mean 4.72 ± 0.98 mg/kg; initially 0.5 mg/kg amphotericin B was diluted in 0.45% NaCl and 2.5% dextrose and administered subcutaneous (SC) route, with a typical frequency of twice weekly for approximately 2 months and then once weekly for an additional 1–2 months before discontinuation. Following amphotericin B discontinuation, fluconazole monotherapy was continued as maintenance. All cats received fluconazole at 10 mg/kg per oral once daily. All cats initially received amphotericin B via the SC route; three cats were switched to intravenous (IV) administration (0.25 mg/kg amphotericin B diluted in 5% dextrose in water) due to development of a sterile abscess at the SC injection site.

Most cases were diagnosed solely by cytology (58.7%). Other diagnostic methods included cytology combined with CALAS (24.3%), CALAS alone (5.2%), CALAS with biopsy (3.9%), CALAS with cytology and biopsy (3.5%), and other combinations such as culture with cytology, LFA, or CALAS in small numbers. Only one case (0.4%) was diagnosed using LFA alone.

Retrovirus testing (FeLV/FIV) was performed in 121 cats (52.6%). Among these, 25 cats (20.7%) tested positive for FeLV, 9 (7.4%) for FIV, and 6 (5%) for both viruses. A total of 81 cats (66.9% of tested cats) were negative. The remaining 109 cats (47.4%) were not tested for retroviruses.

Regarding clinical forms, the nasal form was most common, found in 137 cats (60%). Of these, 17 cats (12.4% of nasal form) also had lymph node involvement. The cutaneous form was observed in 54 cats (23.5%), of whom 14 had lymph node involvement. Disseminated form was diagnosed in 35 cats (15.2%), including combinations such as nasal and cutaneous, CNS and cutaneous, or lung involvement. The CNS form alone was rare (1.3%), as was the ocular form (0.4%). Among cats with lymph node involvement, some were also FeLV or FIV positive (Table 2).

**Figure 2:**
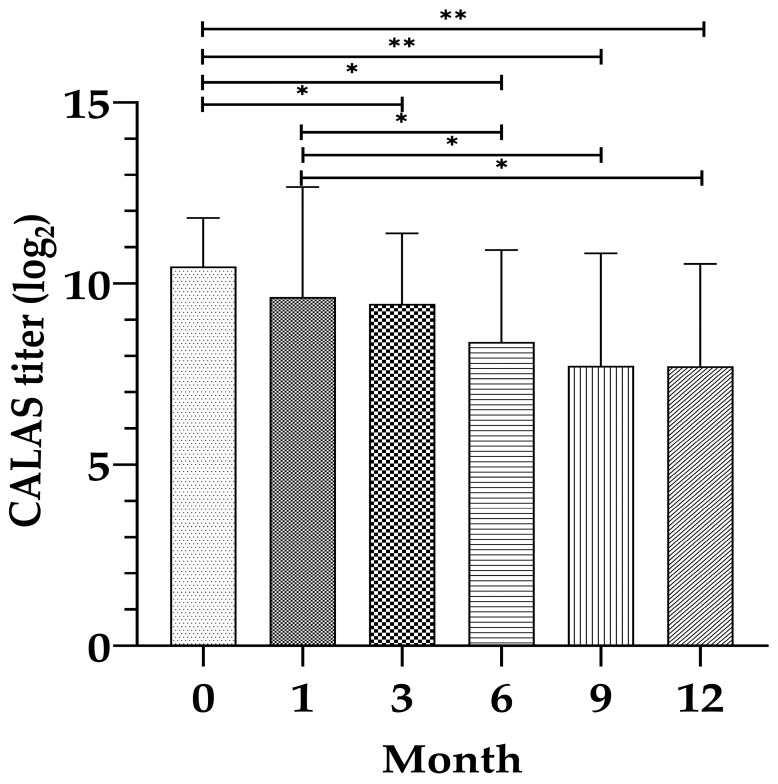
Mean ± SD of CALAS titers (log_2_ scale) in cats with cryptococcosis at months 0, 1, 3, 6, 9, and 12. There was a significant decrease in titers over time. Asterisks indicate significant differences between timepoints (* *p* < 0.05, ** *p* < 0.01).

**Figure 3:**
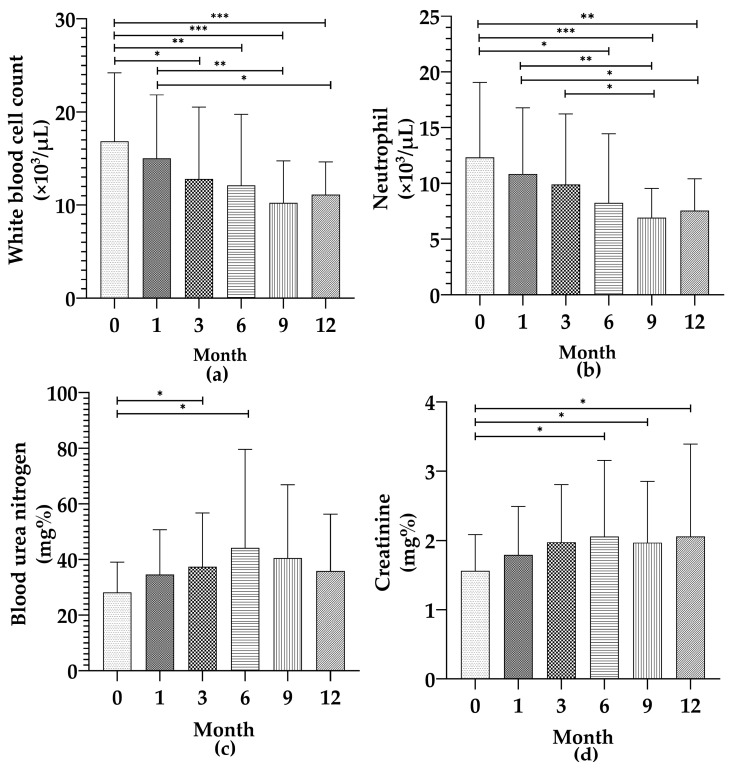
Changes in selected blood parameters during treatment in 35 cats with cryptococcosis over 12 months. (**a**) Mean ± SD of total white blood cell count (×10^3^/µL) at months 0, 1, 3, 6, 9, and 12. (**b**) Mean ± SD of neutrophil count (×10^3^/µL) at months 0, 1, 3, 6, 9, and 12. (**c**) Mean ± SD of blood urea nitrogen (BUN) levels (mg/dL) at months 0, 1, 3, 6, 9, and 12. (**d**) Mean ± SD of serum creatinine levels (mg/dL) at months 0, 1, 3, 6, 9, and 12. Asterisks indicate statistically significant differences between time points (* *p* < 0.05, ** *p* < 0.01, *** *p* < 0.001).

For analysis of sequential clinical changes and treatment response, A subgroup of 35 cats with monthly follow-up from baseline to month 12 was analyzed (Table 3). The CALAS titers were log_2_-transformed before statistical analysis. A significant decrease in titers over time (*p* = 0.001) was observed, with mean log_2_-titers decreasing from 12.00 at baseline to 6.79 at month 12 (Figure 2). WBC also showed a statistically significant decrease (*p* = 0.001), from a baseline mean of 16.85 × 10^9^/L to 10.90 × 10^9^/L at month 12 (Figure 3). Among leukocyte subsets, neutrophils significantly decreased (*p* = 0.001), from 13.33 × 10^9^/µL at baseline to 7.56 × 10^9^/µL at month 12 (Figure 3), while lymphocytes, monocytes, and eosinophils did not show significant changes (*p* = 0.99, 0.44, and 0.54, respectively). The NLR showed a non-significant downward trend from 6.60 at baseline to 3.18 at month 12 (*p* = 0.14).

Red blood cell indices, including RBC and HGB, remained stable over time (RBC: *p* = 0.81; HGB: *p* = 0.75). Mean corpuscular indices were likewise stable: MCV changed minimally from 46.75 to 46.36 fL (*p* = 0.94), MCH from 15.66 to 15.92 pg (*p* = 0.99), and MCHC from 33.86 to 34.50 g/dL (*p* = 0.64).

BUN levels significantly increased over time (*p* = 0.04), with the highest mean value of 43.58 mg/dL occurring at month 6, followed by a slight decrease to 35.41 mg/dL at month 12 (Figure 3). Similarly, serum creatinine levels increased significantly from 1.56 mg/dL at baseline to 2.11 mg/dL at month 12 (*p* = 0.04) (Figure 3). In contrast, no significant differences were observed for ALT (*p* = 0.56), HCT (*p* = 0.50), or PLT (*p* = 0.38), indicating that these parameters remained relatively stable throughout the treatment course.

**Figure 4:**
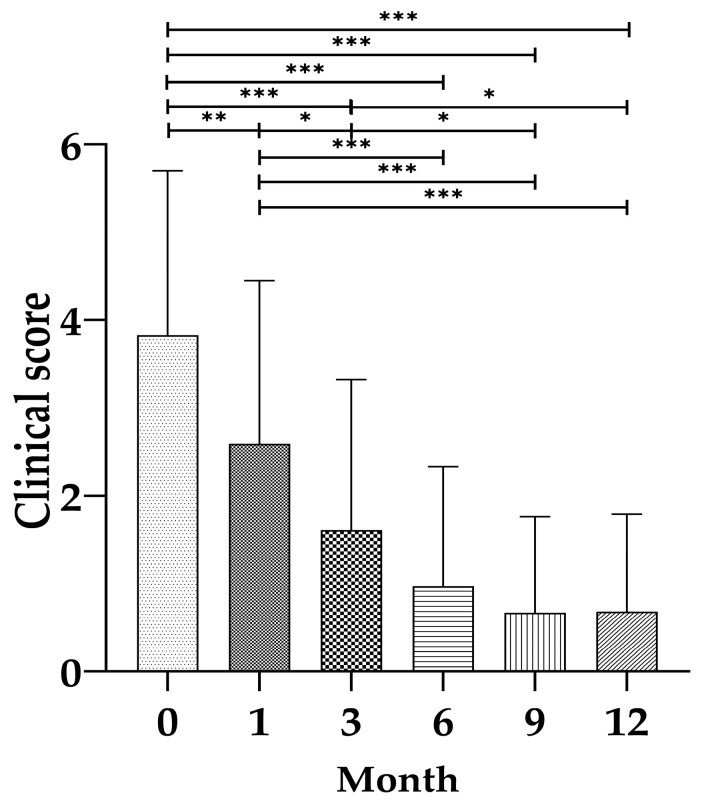
Changes in clinical scores over time in 35 cats with cryptococcosis. Mean ± SD of total clinical score at months 0, 1, 3, 6, 9, and 12 after initiation of antifungal treatment. Asterisks indicate statistically significant differences between timepoints (* *p* < 0.05, ** *p* < 0.01, *** *p* < 0.001).

A statistically significant improvement in clinical score was observed over the 12-month treatment period (*p* < 0.001). The estimated mean clinical score declined steadily from 3.83 at baseline (month 0) to 0.68 at month 12. These findings suggest progressive clinical resolution over time following antifungal therapy (Figure 4).

## 4. Discussion

In this 10-year, single-center study of 230 cats with cryptococcosis, we found that the signalment and pattern of organ involvement at a Thai referral hospital were similar to those reported in Australia and North America. Most affected cats were young to middle-aged Shorthairs, and the nasal or upper-respiratory form was the most common. Many of these cats presented with chronic sneezing, nasal discharge, facial swelling, or nasal-bridge deformity, all of which are typical signs of upper-respiratory cryptococcosis. Cutaneous, disseminated, CNS, and ocular forms were less common and were usually seen in cats with more severe or long-standing disease. These findings agree with extensive studies from Australia and other regions, where nasal disease is usually the main presentation and CNS involvement is less frequent but associated with a worse prognosis when it occurs [9,18,19,20,21,32]. Our results suggest that, even in a tropical Asian country, the clinical picture of feline cryptococcosis is similar to that observed in temperate countries. This means that clinicians in Thailand can apply many of the same diagnostic and treatment principles reported in Australian and North American cohorts, while remaining aware that Cryptococcus species and genotypes may differ and could contribute to subtle differences in severity, relapse risk, or treatment response [11,16,21,27,33,34,35,36].

The high number of nasal and upper respiratory cases in our study supports the idea that cats are mainly infected by inhaling *Cryptococcus* from the environment. The fungus can be found in soil, bird droppings, and decaying wood or trees [6,7,8,11,16]. The identification of several species within the *C. neoformans/gattii* complex has revealed differences in preferred hosts, environmental niches, and disease patterns. For example, *C. gattii* often infects immunocompetent hosts and is linked with severe CNS disease, especially in tropical and subtropical areas [16,27,36]. The Vancouver Island outbreak, caused by a rare *C. gattii* genotype, is a clear example of how environmental changes can drive clusters of disease in both humans and animals [15,35]. We did not routinely perform genotyping in our cases. However, the similarity of our clinical forms to those reported in Australia and North America suggests that both *C. neoformans* and *C. gattii* probably occur in Thailand, and that cats may act as sentinels for environmental risk, as proposed in other endemic regions [2,8,15,21,33,34,35].

CNS and ocular involvement were relatively uncommon in our population, but when present, they were associated with high morbidity, possible long-term neurological deficits, and a guarded prognosis [20,21,27,28,32,37]. Studies from Australia and California report that cats and dogs with CNS cryptococcosis can exhibit a range of neurological signs, characteristic MRI or CT lesions, and variable outcomes depending on lesion type and location [20,32]. Ocular disease was rare in our cats, but the veterinary pathology literature describes granulomatous chorioretinitis, optic neuritis, and panophthalmitis in systemic mycoses, including cryptococcosis [32,37]. A recent immunopathological study of systemic infectious diseases in cats showed that ocular lesions are influenced by local inflammation, vascular damage, and fungal invasion. It may progress even when systemic signs improve [37]. These data support careful neurological and ophthalmic examinations at diagnosis and follow-up, especially in cats with severe or disseminated diseases.

Retroviral infections (FeLV and FIV) can weaken cell-mediated immunity, so they may change the risk and outcome of cryptococcosis [1,7]. In our data, retroviral status was known for 121/230 cats (FeLV 10.9%, FIV 3.9%, both 2.6%, negative 35.2%), but 47.4% were unknown; testing was also not recorded consistently in the 12-month subset (*n* = 35). Because many results were missing and testing was not uniform, we did not compare outcomes by FeLV/FIV status. Prior reports show mixed findings: a large Australian series did not find a consistent link between retroviral infection and specific cryptococcal features [9], while guidelines and reviews note possible worse outcomes in some FeLV/FIV-positive cats [1,7].

A key strength of this study is the combined evaluation of serial CALAS titers, routine clinicopathology, and a clinical score over 12 months in 35 cats. We found that log_2_ CALAS titers decreased over time, in parallel with better clinical scores and reductions in leukocytosis and neutrophilia, while HCT, red blood cell indices, and ALT stayed largely stable. BUN and creatinine increased in some cats. Overall, this pattern suggests that antigen burden, systemic inflammation, and clinical condition usually move in the same direction during successful treatment. This agrees with earlier feline studies, which found that declining serum titers were associated with clinical improvement and eventual cure [5,19]. In human cryptococcal meningitis, serum antigen detection is central to diagnosis and risk stratification, whereas culture-based measures of fungal clearance correlate most strongly with outcome [23,28,37,38]. In many veterinary settings, culture and repeated CSF sampling are complex. Our findings indicate that, in cats, serial serum antigen titers remain a practical way to monitor response when interpreted together with clinical scores and routine blood tests [22,23,28,29,30,38,39].

In our feline cohort, the use of amphotericin B plus fluconazole is similar to human and veterinary practice, where flucytosine cannot be used, and is also consistent with feline guidelines that recommend amphotericin B or azole-based protocols, sometimes with other drugs, for severe or disseminated cryptococcosis [1,7,22,25,30,38,40]. Recent clinical trials in humans give more context for our treatment choices. A large, randomized trial showed that a single high-dose infusion of liposomal amphotericin B, combined with oral flucytosine and fluconazole, can be as effective for HIV-associated cryptococcal meningitis and cause fewer side effects [31]. These results suggest that, in veterinary medicine, liposomal amphotericin B may reduce kidney damage when available and affordable [29,30,31,38,40].

Regarding renal outcomes, only BUN and creatinine were systematically collected; urine specific gravity (USG) and symmetric dimethylarginine (SDMA) were not available. Consequently, we could not reliably distinguish pre-renal azotemia (e.g., dehydration) from intrinsic nephrotoxicity in all cases. In this cohort, azotemia occurred only among cats treated via the SC route; none of the cats that received IV amphotericin B developed azotemia. However, the IV subgroup was small (*n* = 3), which limits route-specific inference and raises the possibility of selection effects (e.g., IV reserved for SC-intolerant cats) and unmeasured confounders such as hydration protocols, electrolyte supplementation, and co-medications. Nephrotoxicity has a significant adverse effect on cats in this study. Seven of 35 cats developed azotemia during amphotericin B treatment. The drug was discontinued in these cats; kidney values returned to normal in 3 of them, but four remained azotemic at the end of follow-up. This is in line with the well-known dose-related kidney toxicity of conventional amphotericin B in both humans and animals [23,25,28,29,30,31,38]. It induces oxidative kidney injury via upregulation of pro-inflammatory cytokines (interleukin-6 and tumor necrosis factor-alpha), inhibits sodium–potassium ATPases and proton exchange, causing renal tubular acidosis and increased tubular permeability, and triggers afferent glomerular arteriolar vasoconstriction. These changes reduce renal blood flow and distal tubular perfusion, leading to a reversible decrease in glomerular filtration rate [41]. Human guidelines emphasize pre-treatment IV fluids, electrolyte correction, and close monitoring of renal parameters and favor liposomal or lipid-complex formulations when possible [28,29,30,31,38]. USG and SDMA were not available, and we did not screen to exclude IRIS Stage 1–2 chronic kidney disease (CKD) before amphotericin B therapy [42]. Therefore, we cannot tell whether the azotemia observed was due to direct amphotericin B nephrotoxicity, pre-renal dehydration, or increased susceptibility from pre-existing renal disease. Our data support similar precautions in cats, including baseline renal evaluation, regular monitoring during therapy, and prompt dose adjustment or discontinuation when azotemia develops [22,25,28,29,30,31,40]. While future work should standardize route choice and renal-safety bundles and include USG and SDMA at predefined intervals to clarify mechanisms and risk factors.

Antifungal susceptibility and genetic diversity of the Cryptococcus isolate from companion animals may also affect the outcome. A North American study found broad susceptibility to common antifungals, notable genotypic diversity, and some *C. gattii* strains with reduced azole susceptibility [33]. This may be important for countries like Thailand, where the ratio of *C. neoformans* to *C. gattii* is poorly understood and where long-term azole monotherapy is standard after induction [7,10,11,21,27,33,40]. Future work in our population should therefore aim to include culture and molecular typing of isolates and link these data with clinical form, antigen kinetics, and treatment response.

Our six-domain clinical score was practical for clinical application and showed a significant decrease over time. This change matched the fall in antigen titers and the improvement in inflammatory markers. Composite scores are widely used in human neurology and infectious diseases, for example, Glasgow Coma Scale-based systems and cryptococcal meningitis severity scores, but they are less well developed for feline cryptococcosis [27,28,29,30,32]. Our score includes organ-specific domains (nasal, CNS, ocular, skin/subcutaneous, systemic, and lymph node involvement). This structure is similar in concept to human guidelines that consider organ involvement and host status when deciding treatment intensity and duration [28,29,30,38]. In practice, our score may help clinicians quantify disease burden, document response between visits, and identify cats whose clinical course does not match their serologic or laboratory results, suggesting the need to look for complications, poor drug penetration, resistance, or issues with owner compliance.

This study has several limitations. It was a retrospective, single-center study with non-standardized follow-up intervals and loss to follow-up. Only 35 of 230 cats met the criteria and could be included in the sequential analysis, reducing statistical power and increasing the risk of missing fundamental differences (type II error). In addition, selection and survivorship biases are likely: restricting the longitudinal analysis to cats with sufficient CALAS titers and sustained follow-up may overrepresent animals that survived early critical phases and owners able to maintain prolonged treatment, which can bias outcomes toward a better prognosis. Disease stage at presentation, previous treatments, and total amphotericin B dose differed between cats and may have influenced blood results and kidney outcomes. Species-level identification (*C. neoformans* vs. *C. gattii*), antifungal susceptibility testing was not consistently performed; therefore, any species-specific comparisons in the discussion should be interpreted as theoretical context, and we cannot infer species-specific virulence or drug response in this cohort. Advanced imaging was not consistently performed, so we could not fully link host factors, pathogen features, and treatment response. In addition, our clinical score, although practical and intuitive, has not yet been externally validated or tested for inter-observer agreement. Future studies should be prospective and, ideally, multicenter, with fixed time points for CALAS testing, clinicopathology, and clinical scoring. They should also include molecular typing of isolates and formal validation of the score against long-term outcomes and quality of life [28,29,30,31,32,43]. Such work would improve prognostic tools, guide treatment decisions, and clarify how best to combine serologic, clinical, and imaging data in the management of feline cryptococcosis.

## Figures and Tables

**Table 1:** Proposed clinical severity score for feline cryptococcosis.

System	Score 0	Score 1 (Mild)	Score 2 (Moderate)	Score 3 (Severe)
Nasal/Upper respiratory	No nasal signs.	Intermittent, mild serous/mucoid nasal discharge; no facial deformity; no dyspnea; appetite normal.	Persistent mucopurulent or bloody discharge, nasal swelling or intranasal mass, stertor or mild dyspnea, but able to eat and drink.	Marked nasal obstruction with open-mouth breathing, severe facial deformity, or inability to eat/drink normally.
CNS	No neurologic abnormalities.	Mild deficits not interfering with ambulation or activities of daily living (e.g., mild ataxia, subtle cranial nerve deficit, mild behavior change).	Apparent neurologic deficits affecting function (e.g., paresis/ataxia impacting gait, unilateral blindness, circling, head tilt, seizures controlled with an anticonvulsant); the cat remains ambulatory.	Life-threatening CNS signs such as status epilepticus or frequent cluster seizures, stupor/coma, non-ambulatory paresis/paralysis, or evidence of severe increased intracranial pressure requiring intensive care.
Ocular	No ocular lesions.	Mild anterior uveitis, conjunctivitis, or chorioretinitis; vision preserved in both eyes.	Severe uveitis or chorioretinitis with vision loss in one eye, retinal detachment, marked ocular discomfort, or optic neuritis in one eye.	Bilateral vision loss (blindness), severe panophthalmitis, glaucoma, or globe rupture/phthisis bulbi.
Cutaneous/Subcutaneous	No skin or subcutaneous lesions attributable to cryptococcosis.	One or two small, non-ulcerated nodules or papules (<1 cm), non-painful, no functional impact.	Multiple nodules or plaques and/or ulceration with exudate; mild local pain or pruritus, but no significant disfigurement.	Extensive or disfiguring lesions (e.g., large facial masses, widespread ulceration), marked pain or secondary infection, or interference with normal function (eating, grooming).
Systemic	Normal body condition, normal appetite and activity, afebrile.	Mild weight loss (<5%), intermittent hyporexia, mild lethargy but still interactive; afebrile or transient low-grade fever (<103 °F).	Moderate weight loss (5–10%) or clearly reduced body condition; persistent hyporexia/anorexia > 48 h; intermittent fever ≥ 103 °F; mild dehydration.	Severe weight loss/cachexia (>10%) or BCS ≤ 3/9; persistent anorexia; sustained high fever; hypotension or moderate–severe dehydration.
Lymph node involvement	No peripheral or internal lymphadenomegaly attributable to cryptococcosis.	Mild enlargement of a single lymph node region (e.g., mandibular or popliteal), non-painful, no ulceration.	Marked enlargement of one lymph node region or involvement of ≥2 regions; nodes may be firm or irregular, with or without mild discomfort; imaging may show enlarged internal lymph nodes without significant compression of adjacent structures.	Generalized or severe lymphadenomegaly with functional impact (e.g., mediastinal or abdominal lymph nodes causing respiratory distress, dysphagia, or other compression signs), and/or lymph node necrosis or ulceration.

CNS: Central nervous system, BCS: Body condition score.

**Table 2:** Demographic and diagnostic characteristics of 230 cats diagnosed with cryptococcosis at Kasetsart University Veterinary Teaching Hospital (2014–2023). The table summarizes sex, breed distribution, diagnostic methods used, retroviral status, and clinical forms based on organ involvement.

Demographic and Diagnostic Characteristics	Percentage
Sex	
Female	53.9% (124/230)
Male	46.1% (106/230)
Breed	
Domestic Shorthair	94.78% (218/230)
Persian	3.04% (7/230)
Scottish Fold	1.3% (3/230)
Abyssinian	0.4% (1/230)
American Wirehair	0.4% (1/230)
Retroviral status	
FeLV infection	10.9% (25/230)
FIV infection	3.9% (9/230)
Both FeLV and FIV infection	2.6% (6/230)
Negative	35.2% (81/230)
Unknown	47.4% (109/230)
Diagnosis	
Cytology only	58.7% (135/230)
Lateral flow assay only	0.4% (1/230)
CALAS only	5.2% (12/230)
Culture only	0.9% (2/230)
Cytology combined with CALAS	24.3% (56/230)
Cytology combined with LFA and Biopsy	3.5% (8/230)
Cytology combined with LFA and culture	1.7% (4/230)
Culture combined with cytology and LFA	0.4% (1/230)
Culture combined with cytology	0.4% (1/230)
Culture combined with LFA	0.4% (1/230)
Lateral flow assay combined with Biopsy	3.9% (9/230)
Clinical form	
Nasal	60% (137/230)
With lymphadenopathy	12.4% (17/137)
Cutaneous	23.5% (54/230)
With lymphadenopathy	26% (14/54)
CNS	1.3% (3/230)
Ocular	15.2% (35/230)
Disseminated	0.4% (1/230)
CNS with ocular and lymphadenopathy	0.4% (1/230)
CNS with cutaneous	0.4% (1/230)
CNS with cutaneous, lower respiratory, and lymphadenopathy	0.4% (1/230)
Nasal with cutaneous	6.4% (14/230)
With lymphadenopathy	14.2% (2/14)
Nasal with ocular	0.4% (1/230)
Gastrointestinal	0.4% (1/230)
Lymphadenopathy	6.9% (16/230)

FeLV: Feline leukemia virus, FIV: Feline immunodeficiency virus, CALAS: Cryptococcal antigen latex agglutination system, LFA: Lateral flow assay, CNS: Central nervous system.

**Table 3:** Mean ± SD of clinical, laboratory parameters, and clinical score at each time point (Month 0, 1, 3, 6, 9, and 12). Statistically significant differences were observed across months in serum antibody titer (log_2_), WBC, neutrophil, BUN, serum creatinine, and clinical score (*p* < 0.05). In contrast, no significant changes were detected in HCT, HGB, lymphocyte, monocyte, eosinophil, basophil, NLR, PLT, or ALT (*p* > 0.05).

Parameter	Month						*p* Value
	0	1	3	6	9	12	
Log_2_-Titer	10.47 ± 0.40	9.63 ± 0.62	8.77 ± 0.59	7.80 ± 0.61	7.42 ± 0.68	7.31 ± 0.75	0.001 **
HCT (%)	34.97 ± 0.72	34.83 ± 0.78	33.38 ± 0.81	35.56 ± 0.75	34.35 ± 0.93	35.14 ± 0.91	0.5
MCV (fL)	46.75 ± 0.86	47.35 ± 0.89	46.24 ± 1.05	46.03 ± 0.99	46.39 ± 1.50	46.36 ± 1.54	0.94
MCH (pg)	15.66 ± 0.28	15.88 ± 0.22	15.75 ± 0.31	15.68 ± 0.32	15.80 ± 0.35	15.92 ± 0.42	0.99
MCHC (g/dL)	33.86 ± 0.33	33.67 ± 0.35	34.15 ± 0.31	34.10 ± 0.40	34.31 ± 0.51	34.50 ± 0.36	0.64
RBC (×10^6^/μL)	7.63 ± 0.27	7.41 ± 0.33	7.17 ± 0.36	7.79 ± 0.31	7.36 ± 0.35	7.59 ± 0.41	0.81
HGB (g/dL)	12.00 ± 0.38	11.58 ± 0.47	11.23 ± 0.53	12.07 ± 0.46	11.40 ± 0.45	11.86 ± 0.58	0.75
WBC (×10^3^/μL)	16.85 ± 1.24	15.02 ± 1.27	12.79 ± 1.44	12.09 ± 1.35	10.23 ± 0.98	11.13 ± 0.77	0.001 **
Neutrophil (×10^3^/μL)	12.33 ± 1.14	10.85 ± 1.10	9.91 ± 1.18	8.43 ± 1.18	6.93 ± 0.60	7.56 ± 0.64	0.001 **
Lymphocyte (×10^3^/μL)	3.03 ± 0.33	3.23 ± 0.43	3.18 ± 0.50	3.30 ± 0.50	2.81 ± 0.57	3.07 ± 0.34	0.99
NLR	6.60 ± 1.55	5.20 ± 0.95	8.32 ± 3.35	4.89 ± 1.69	3.65 ± 0.48	3.18 ± 0.58	0.14
Monocyte (×10^3^/μL)	0.59 ± 0.20	0.40 ± 0.08	0.28 ± 0.09	0.25 ± 0.06	0.25 ± 0.08	0.24 ± 0.08	0.44
Eosinophil (×10^3^/μL)	0.87 ± 0.16	0.57 ± 0.09	0.54 ± 0.10	0.54 ± 0.09	0.58 ± 0.14	0.50 ± 0.11	0.54
Basophil (×10^3^/μL)	0.002± 0.005	0.004 ± 0.018	0 ± 0	0.001 ± 0.004	0.001 ± 0.002	0.005 ± 0.02	0.34
PLT (×10^3^/μL)	202.85 ± 12.96	163.17 ± 14.23	177.61 ± 14.23	185.98 ± 13.55	173.21 ± 16.73	197.26 ± 16.73	0.38
BUN (mg%)	28.19 ± 1.94	34.54 ± 3.17	37.44 ± 3.86	44.13 ± 6.37	40.50 ± 6.22	35.80 ± 4.59	0.04 *
Creatinine (mg%)	1.56 ± 0.09	1.79 ± 0.13	1.98 ± 0.15	2.06 ± 0.19	1.99 ± 0.17	2.06 ± 0.25	0.04 *
ALT (U/L)	73.51 ± 9.76	69.92 ± 10.92	48.30 ± 10.37	57.80 ± 10.21	68.48 ± 11.55	66.32 ± 11.55	0.56
Clinical Score	3.83 ± 0.32	2.59 ± 0.32	1.61 ± 0.30	0.97 ± 0.23	0.67 ± 0.20	0.68 ± 0.22	<0.001 ***

HCT: hematocrit, MCV: mean corpuscular volume, MCH: mean corpuscular hemoglobin, MCHC: mean corpuscular hemoglobin concentration, RBC: red blood cell count, HGB: hemoglobin concentration, WBC: white blood cell count, NLR: neutrophil–lymphocyte ratio, PLT: platelet count, BUN: blood urea nitrogen, ALT: alanine aminotransferase; * *p* < 0.05, ** *p* < 0.01, *** *p* < 0.001.

## Data Availability

The original contributions presented in this study are included in the article. Further inquiries can be directed to the corresponding author.
